# Cardiac Thromboembolism in COVID-19: A Case Series

**DOI:** 10.7759/cureus.25193

**Published:** 2022-05-21

**Authors:** Sachin Kumar, Sanchit Chawla, Hussain Karimi, Taha Ahmed, Gautam Shah

**Affiliations:** 1 Department of Internal Medicine, Fairview Hospital - Cleveland Clinic, Cleveland, USA; 2 Department of Hospital Medicine, University of Kentucky, Lexington, USA; 3 Department of Cardiology, Fairview Hospital - Cleveland Clinic, Cleveland, USA

**Keywords:** aspiration thrombectomy, biventricular thrombus, lad thrombus, covid hypercoagulability, st-elevation myocardial infarction (stemi)

## Abstract

Venous thromboembolism (VTE) is a very common complication of coronavirus disease-2019 (COVID-19) because of the acquired hypercoagulability in these patients. Cardiovascular thromboembolism (CTE) is another complication that is relatively rare yet catastrophic. We present two cases of COVID-19 which were complicated by CTE. Case one describes a 55-year-old male with COVID-19 who had an ST-segment elevation myocardial infarction (STEMI) secondary to coronary artery embolism and was also found to have biventricular thrombi (BVT). Case two describes a 65-year-old female presenting with STEMI secondary to coronary artery embolism. This document highlights how CTE can be present in COVID-19 patients and describes the available evidence for its management. Given the paucity of data on these complications, we illustrate the offered treatment which was based on the data extrapolated from the treatment of VTE in COVID-19 and the treatment of CTE in non-COVID-19 patients.

## Introduction

As of March 2022, about 487 million individuals have had COVID-19, which has led to approximately six million fatalities worldwide. The disease spectrum varies from asymptomatic presentation to severe degrees of organ failure and death. Since COVID-19 activates all three components of Virchow's triad, thromboembolic complications are common and frequently manifest as deep vein thrombosis, pulmonary embolism, or acute stroke [1]. Cardiac manifestations of COVID-19 such as acute coronary syndrome, myocarditis, and arrhythmias are known to be associated with increased all-cause mortality and worse outcomes [2]. Cardiovascular thromboembolism (CTE) is an infrequent yet life-threatening complication of COVID-19 infection [2]. Unlike venous thromboembolism (VTE), available data regarding the frequency and management of CTE is limited and there needs to be increased awareness for early diagnosis and treatment that can help improve the morbidity and mortality of these patients.

## Case presentation

Case one

A 55-year-old male presented to the emergency department with fever, chills, malaise, and shortness of breath which started seven days before presentation. The patient had no significant past history. Initial blood pressure was 135/92 mmHg, heart rate was 78 beats per minute, temperature was 99.6, respiratory rate was 20/min, and oxygen saturation on room air was 90%, requiring him to wear 2 L/min of supplemental oxygen.

Patient tested positive for COVID-19. Electrocardiogram was normal. On lab investigations, d-dimer was 1,250 ng/mL (normal <500 ng/mL), troponin-T was <6 ng/mL (normal <0.4ng/mL), proBNP was 142 pg/mL (normal <100 pg/mL), prothrombin-time was 10.5s (normal 11-13.5s), international-normalized-ratio was 1.0 (normal <1.1), and partial-thromboplastin-time was 23.9s (normal 21-35s). A computed-tomography (CT) angiography of the chest showed multifocal bilateral ground glass opacities with no pulmonary embolism.

**Figure 1:**
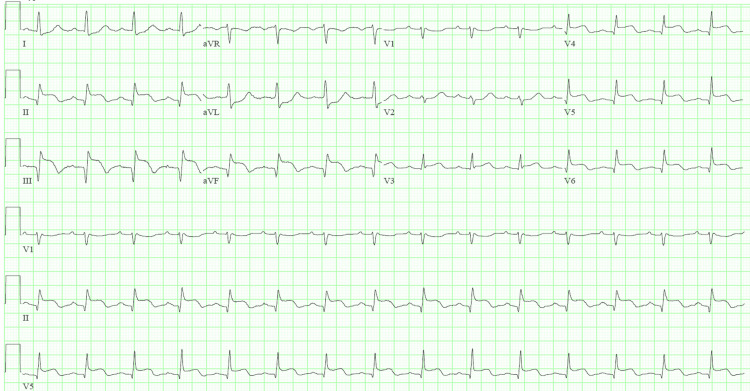
Case one electrocardiogram showing ST-elevation ST-segment elevation in lead II, III, and aVF; consistent with inferior wall myocardial infarction

**Figure 2:**
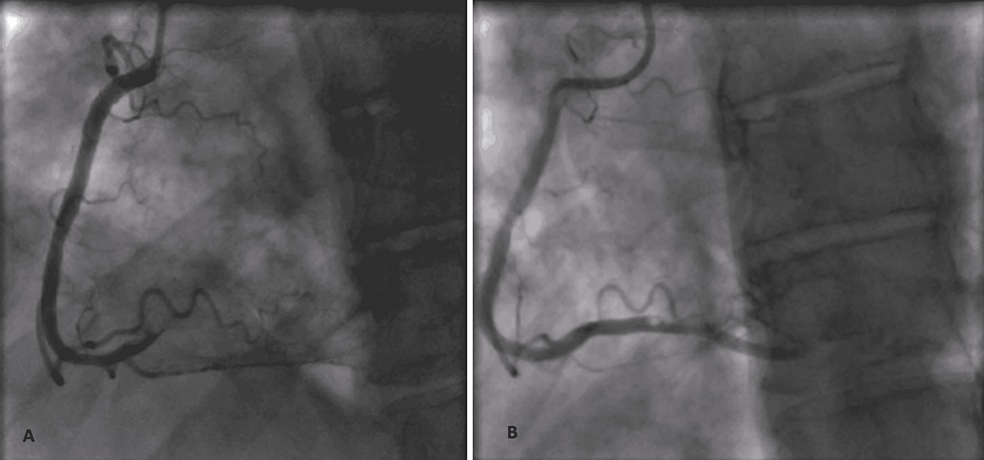
Case one coronary angiogram (A) The mid-vessel occlusion of RCA with the filling defect. (B) Improvement of the RCA occlusion with antegrade flow and evidence of a significant residual luminal filling defect. RCA: right coronary artery.

**Figure 3:**
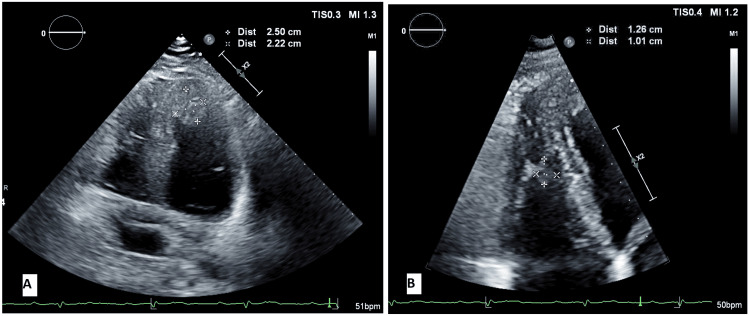
Case one echocardiogram showing biventricular thrombi (A) The left ventricular thrombus measuring 2.50 x 2.22 cm and (B) The right ventricular thrombus measuring 1.26 x 1.01 cm.

The patient was treated with dexamethasone 6 mg, remdesivir 100 mg (following the 200 mg loading dose), and enoxaparin 40 mg daily. On day 4 of hospitalization, he complained of severe upper back and chest pain. Electrocardiogram revealed new ST-segment elevations in inferior and lateral leads (Figure 1). ST-segment elevation myocardial infarction (STEMI) was diagnosed and the patient was given aspirin and intravenous heparin. The patient was emergently taken for cardiac catheterization, which revealed mid-vessel occlusion of the right coronary artery (Figure 2) without evidence of an atherosclerotic component. Other coronary arteries had no stenosis. Percutaneous transluminal balloon angioplasty of the right coronary artery was performed. This was followed by aspiration thrombectomy and despite six attempts of large volume thrombus aspiration, only thrombolysis in myocardial infarction (TIMI) 1 flow was achieved (Figure 2). Tirofiban was administered given the high thrombus burden. Post-procedure, the patient developed acute left-sided hemiplegia with right-sided gaze deviation. Tirofiban was discontinued and an emergent CT head was performed which revealed complete occlusion of the right middle cerebral artery. The patient underwent cerebral artery thrombectomy, following which his focal neurological deficits improved significantly. Aspirin 81 mg and atorvastatin 80 mg were continued. Clopidogrel and beta-blocker were held in the setting of a recent large stroke. An echocardiogram following these events showed moderately decreased left ventricular systolic function, a large left ventricular thrombus measuring 2.50 x 2.22 cm, and a large right ventricular mobile thrombus up to 2.17 cm (Figure 3). The patient was started on heparin bridge to warfarin.

Three days post-STEMI, the patient was noted to have intermittent complete heart block (CHB). Respecting the patient’s wishes, intermittent nature of the CHB, stable escape rhythm, and presence of right ventricular thrombus; a decision was made to not insert a pacemaker. Two days later, no more episodes of CHB were noted.

Following these events, the patient improved, remained afebrile, and was weaned off supplemental oxygen. His CHB resolved, and his only neurological deficit was a left facial droop. He was discharged on aspirin, atorvastatin, and warfarin.

Case two

A 65-year-old female presented to the emergency department with acute onset chest pain. Ten days prior to this, she was diagnosed with COVID-19, at which time her only symptoms were myalgias and non-productive cough which resolved within a week. She also had a past medical history of hyperlipidemia, osteopenia, chronic gastritis, and gastroesophageal reflux disease. She denied any associated cough, shortness of breath, or fever at the time of presentation. In the emergency department, her blood pressure was 129/79 mmHg, heart rate was 89 beats per minute, temperature was 97.9, and oxygen saturation on room air was 96%.

**Figure 4:**
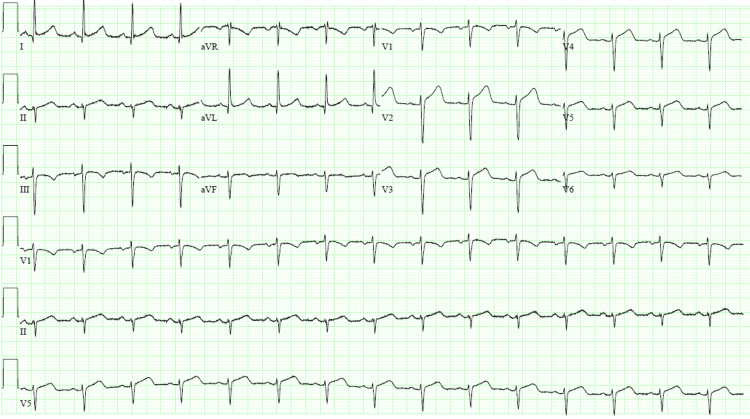
Case two electrocardiogram showing ST-elevation ST-segment elevation in lead V2, V3, V4, V5, and V6; consistent with anterior wall myocardial infarction.

**Figure 5:**
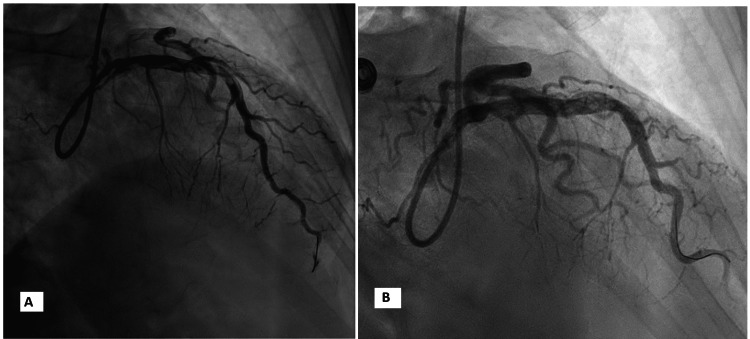
Case two coronary angiogram (A) A LAD thrombus with the filling defect seen on the right oblique view. (B) The left oblique cranial view showing the LAD post-intervention. LAD: left anterior descending

An electrocardiogram showed sinus rhythm with ST-segment elevations in anterior leads (Figure 4). Her troponin-T was 0.950 ng/mL. The patient was diagnosed with STEMI. Cardiac catheterization revealed a large clot in the left anterior descending artery and no evidence of coronary atherosclerosis (Figure 5). No stenosis was found in other coronary arteries. Aspiration thrombectomy was performed and TIMI 3 flow was restored. She was treated with apixaban 10 mg twice daily for 10 days followed by five mg twice a day for three months.

Outcome and follow-up

Case one was lost to follow up. Regarding case two, after three months, apixaban was discontinued and a hypercoagulation workup was performed, which was unremarkable. This workup included prothrombin-time, international-normalized-ratio, partial-thromboplastin-time, fibrinogen, thrombin time, activated protein C resistance, factor VIII:C assay, protein-c, protein-s function, antithrombin assay, and hexagonal (II) phase phospholipid neutralization assay.

## Discussion

VTE is a well-established complication of COVID-19 [3]. CTE is an infrequent complication, amidst which acute coronary embolism causing STEMI and ventricular thrombi are rare but catastrophic complications of COVID-19-related hypercoagulability [2,4]. Such events can occur despite adequate anticoagulation prophylaxis [5]. Although there is significant data guiding the prevention and treatment of VTE in COVID-19 [6], there is a paucity of data guiding the management of STEMI and/or biventricular thrombi (BVT) in such patients.

A retrospective observational study looking at 5,119 COVID-19 patients, found that 17 patients had their first-ever MI while having COVID-19, out of which only four had classical risk factors such as diabetes or hypertension. The study also found that 44 patients had their first-ever ischemic stroke while having COVID-19. The incidence of these complications was 5-10 times higher during the first 14 days of COVID-19 diagnosis [4]. Metanalyses have concluded the incidence rates of arterial thromboembolism to be 3%-3.9% in COVID-19 patients [3,7]. Stefanini et al. performed a study looking into the characteristics of 28 COVID-19 patients who had STEMI [8] and found that for 85% of patients, STEMI was the first clinical manifestation of COVID-19. All patients involved in the study underwent coronary angiography which found no culprit lesion in 11 (40%) patients, classifying them as type 2 MI.

Though the pathophysiology behind the COVID-19 hypercoagulability is incompletely understood, COVID-19 is known to affect all three components of Virchow’s triad [1]. The virus interacts with angiotensin-converting-enzyme 2 receptors causing endothelial activation and injury. Infected patients, especially the ones admitted to the hospitals are likely to be immobilized, more so if intubated, causing stasis of blood flow. Also, the affected patients are found to have hyper-viscosity, elevated inflammatory markers, and platelets which contribute to hypercoagulability of the blood [1]. The level of D-dimer is the strongest predictor of the risk of thrombo-embolic events in COVID-19 patients [1].

National Cerebral and Cardiovascular Center criteria proposed by Shibata et al for the diagnosis of coronary embolism have three major and three minor criteria [9]. The three major criteria are 1. angiographic evidence of coronary embolism without atherosclerotic components; 2. concomitant coronary embolization at multiple coronaries; 3. concomitant systemic embolization without left ventricular thrombus attributable to acute myocardial infarction. The three minor criteria are 1. <25% coronary stenosis, except for the culprit lesion; 2. evidence of an embolic source via imaging; 3. the presence of embolic risk factors such as hypercoagulable state, atrial fibrillation, and infective endocarditis among others. The criteria require the presence of ≥ two major criteria, one major criterion plus ≥2 minor criteria, or three minor criteria for the definite diagnosis of coronary embolism. Both the cases meet the first major and first minor criteria given the high thrombus burden without any component of atherosclerosis and absence of stenosis in other coronary arteries except the culprit artery. Given that COVID-19 is an established hypercoagulable disorder [6], both the cases also meet the third minor criteria. Thus, the described two cases meet the criteria for the definite diagnosis of coronary embolism. Whether the left ventricular thrombus in case one was the source of the coronary emboli or a consequence of STEMI secondary to the coronary emboli is difficult to determine in the absence of a pre-STEMI echocardiogram.

Given the absence of guidelines to direct the management of coronary embolism, management decisions are at the treating physician’s discretion [10]. Available options include coronary stent placement with anti-platelet therapy versus anticoagulation versus both. Fibrinolysis and percutaneous coronary intervention, both have been used to manage STEMI in COVID-19 [11,12]. The majority of COVID-19 patients who underwent percutaneous coronary intervention and received a drug-eluting stent in the culprit lesion had a significantly elevated risk of stent thrombosis as compared to non-COVID-19 patients who underwent drug-eluting stent placement [11,12]. There has been an inconsistent use of long-term anticoagulation after revascularization in COVID-19 patients with STEMI [13,14]. Hence, for the described patients, a coronary angiogram was performed and a culprit lesion was identified, which was managed with aspiration thrombectomy, anti-platelet agents, and anticoagulation; no coronary stent was placed.

In the management of left ventricular thrombus, the American College of Cardiology recommends warfarin over direct-oral-anticoagulants (DOAC) for ≥ three months with repeat imaging to ensure resolution of the thrombus [15]. Currently, there are no guidelines or expert consensus available for the treatment of ventricular thrombi in COVID-19. Expert consensus for the management of VTE in COVID-19 recommends the use of either warfarin or DOAC for ≥ three months [6]. So far, only four cases have been reported of BVT in COVID-19, only one patient survived, and was discharged on warfarin [5,16-18]. For the management of the described patients, data was extrapolated from the treatment of VTE in COVID-19 and the treatment of left ventricular thrombus in non-COVID-19 patients [6,15].

## Conclusions

COVID-19-induced hypercoagulability can lead to a myriad of thrombo-embolic complications. It is important for treating physicians to recognize STEMI as a complication of COVID-19 even in the absence of classical risk factors of coronary artery disease. With this case series, we have highlighted atypical causes of STEMI in COVID-19 patients such as coronary artery emboli. Coronary artery embolism and BVT remain rare but fatal complications of COVID-19 despite adequate anticoagulation prophylaxis. Hence, treating physicians should remain vigilant for CTE and related sequelae in these patients. Due to the limited data, there are no guidelines or consensus for the management of CTE in COVID-19 patients. Further research is needed to determine the role of percutaneous coronary interventions, anticoagulation, and antiplatelet agents in these patients.
